# The Possibilities of Using Broadleaf Cattail Seeds (*Typha latifolia L.*) as Super Absorbents for Removing Aromatic Hydrocarbons (BTEX) from an Aqueous Solution

**DOI:** 10.1007/s11270-018-4058-9

**Published:** 2018-12-18

**Authors:** Tomasz Ciesielczuk, Czesława Rosik-Dulewska, Joanna Poluszyńska

**Affiliations:** 1 Institute of Environmental Protection and Development, Opole University, Oleska Str. 22, 45-052 Opole, Poland; 2 Institute of Environmental Engineering of the Polish Academy of Sciences, Skłodowskiej-Curie Str. 34, 41-819 Zabrze, Poland; 3 Institute of Ceramics and Building Materials, Oswiecimska Str. 21, 45-641 Opole, Poland

**Keywords:** Water, BTEX, Sorption, Broadleaf cattail seeds, Mercerization

## Abstract

Sorption of oil-related products (including mainly the propellants) is the very basic process that counteracts spreading these types of pollution into environment. Plenty of synthetic substances (including the monoaromatic hydrocarbons) are both from the surface and underground waters. The aim of this study was to present the research’s results on the possibilities of using the broadleaf cattail (*Typha latifolia L*.) seeds as a sorbent of monoaromatic hydrocarbons from an aqueous solution. In order to increase sorptive capacity, the seeds biomass was submitted for the process of mercerizing in diversified time and temperature in water and the NaOH solution. The removal of benzene, toluene, ethylbenzene, o-xylene, m-xylene and cumene was carried out by means of the “batch method”. All the conducted experiments have shown a high sorption level of the analysed pollutions from an aqueous solution. The best sorptive qualities appeared in the seeds drenched in 80 °C water for 4 h (W) 97 g/kg, what was 9.06% more absorbed hydrocarbons in comparison to the control sample (C) and 26.8% more than the smallest seeds drenched in NaOH for 240 min. in the temperature of 80 °C (N). The process of the seeds mercerizing that was conducted with the use of hot water appeared to be most effective, but seeds without mercerisation (C) is actually the material which absorbs the least amounts of energy for preparation and had quite good sorption capacity too.

## Introduction

To limit the negative effects of gas oil, mazut or petrol spills, great caution has to be taken when dealing with vats that are used for transporting these substances. Nevertheless, as a result of various disasters, both terrestrial and marine, such as accidental overflows at the petrol transshipment stations or damage to transmission lines, the aforementioned spills lead to numerous serious threats which result in the degradation of waters and soils used for agricultural purposes. In such circumstances, sorbents of oil-related pollution are employed for a very wide range of uses (Chen et al. 2008; Möller et al. 2009; Oyanedel-Crever et al. 2007; Półka et al. 2015; Rosik-Dulewska et al. 2012; Włodarczyk-Makuła 2017; Yu et al. 2016). In the case of oil-related pollution, the application of compost from waste can be a good solution as it offers the possibility of reusing the compost as a sorbent after a specific period of time required for the biodegradation of the absorbed compounds (Ciesielczuk 2017; Rosik-Dulewska and Ciesielczuk 2013).

Sorbents should also be characterised by huge sorptivity in differential temperature and humidity conditions; however, the effectiveness of sorption depends on the type of adsorbent applied (mainly on the active surface, and the amount and size of pores), the concentration of the adsorbate, the temperature and humidity, the contact time of the adsorbate and adsorbent as well as the mutual affinity of both of the centres. The most frequently used adsorbents are active carbon, zeolites, colloidal silicon dioxide, aluminogels, metal oxides, fibrous materials of a natural origin and synthetically cross-linked polymers (Dąbek 2015). Naturally complex fibrous sorbents from cellulose, hemicelluloses and xylogen are promising sources of biomass that can be used for sorbent production, especially when taking the residual biomass into consideration (Mwaikambo and Ansell 2002). A large number of sorbents can be regenerated through abstraction, most often via the decomposition of an adsorbate’s molecules due to which it becomes possible to reuse them. In most cases, however, a sorbent that has already been used has to be incapacitated (Ciesielczuk 2017; Kuśmierek et al. 2017; Kyzioł-Komosińska et al. 2011).

Synthetic fibrous materials used as sorbents are primarily products that are generated from polyvinyl chloride (PCV) or polystyrene (PS). Their sorptivity is quite considerable and often exceeds 100 g of an oil-related product in proportion to 1 g of sorbent (1000%). Furthermore, they can be neutralised in combustion plants for dangerous wastes, thus providing the opportunity to regain energy (Wong et al. 2016). Still, other authors pay attention to another option—desorption. This is the removal of the absorbed hydrocarbons through impressing or via a hexane rinse, for example, after which it possible for the sorbent to be used again (Pintor et al. 2016). One way or another, the usage of sorbents that have been used previously to remove organic compounds dissolved in water is extraordinarily problematic. Aromatic hydrocarbons are quite easily soluble and due to that fact, the process of sorption becomes more difficult (Piekutin 2015a, 2015b). What is more, the presence of surfactant character substances can additionally reduce the level of the compounds’ activation on the sorbent’s surface. An easily available biomass, including species that are invasive in many countries (e.g. goldenrod), may be applied as cheap sorbents of oil-related substances (Ciesielczuk et al. 2016).

The broadleaf cattail seeds suggested in this research are easy to source because of the infructescent structure of the plant. Therefore, they can be successfully used as cheap hydrophobic sorbent that is available in different climatic conditions. Using this material for BTEX sorption out of a water compound has not been tested yet; for this reason, the topic is being taken up. The aim of the research was to conduct a trial to test broadleaf cattail seeds (*Typha latifolia L.*) being applied to absorb aromatic hydrocarbons out of a water compound.

## Materials and Methods

The monoaromatic hydrocarbons’ sorption (benzene, toluene, ethylbenzene, o-xylene, p-xylene and cumene, the characteristics of which have been listed in Table 1) was conducted through “batch-type” experiments in a room temperature of 20 ± 2 °C). The sorbents applied in the research were six types of materials produced in the mercerisation of the aspen poplar seed biomass. What is more, all the reagents had a degree of purity appropriate to the chromatographic analysis.

**Table 1:** Basic parameters of investigated compounds

Compound CAS number	Molar mass [g/mol]	Water solubility (20 °C) [mg/dm^3^]	Density [g/cm^3^]	Concentration in adsorbate [mg/dm^3^]
Benzene 71-43-2	78.12	1820	0.8786	331.6
Toluene 108-88-3	92.14	520	0.873	146.8
Ethylbenzene 100-41-4	106.17	150	0.8669	46.2
p-Xylene 1330-20-7	106.17	180	0.861	46.7
o-Xylene 108-38-3	106.17	170	0.8969	48.9
Cumene 98-82-8	120.19	50	0.862	16.3

### The Preparation of Samples

The seed samples were collected and dried to a constant mass at 90 °C. The process of mercerisation was carried out in cold and hot conditions on account of which the following types of seed biomass have been extracted:

- C blank test without mercerisation
- H drenching in distilled water at a temperature of 80 °C for 60 min
- W drenching in distilled water at a temperature of 80 °C for 240 min (Gąsiorowski et al. 2012)
- N drenching in a 5% NaOH solution at a temperature of 80 °C for 240 min (Mohd et al. 2007)
- Na drenching in a 5% NaOH solution at a temperature of 20 °C for 2 days (Nascimento et al. 2018)
- G drenching in standing water at a temperature 20 °C for 15 days (Witka-Jeżewska et al. 2003)

Testing of the hydrocarbons extracted from the water solution was carried out in glass vessels closed with microsection. The water solution was prepared via a process of dissolving hydrocarbons mixed in equal proportions (500 μl)—benzene (B), toluene (T), ethylbenzene (E), o-xylene (O), p-xylene (P) and cumene (C)—in 1000 cm^3^ of redistilled water containing ethylene glycol in the amount of 5 cm^3^/dm^3^. The characteristics of the tested compounds are presented in Table 1. The obtained water solution was kept at a temperature of 20 °C without any light. Each time, about 0.2 g of the aspen poplar seeds were directly weighed out and placed into the vessels. The proportion of sorbent to water solution was 1:200. The amount of the water solution was calculated to obtain an exact 1:200 proportion in each experimental vessel. The sorption time was 15, 30, 60, 120, 240, 360, 720 and 1440 min. After this time, the samples with the solution were taken and then submitted for extraction by means of dichloromethane. They were then shaken in laboratory glasses (4 cm^3^) made from dark glass closed with a Teflon cap. The eluate was dried with Na_2_SO_4,_ which had been previously dried at 130 °C. Each of the experiments was conducted independently three times. Aromatic hydrocarbons were marked in untreated extracts (or in blank samples) by the GC-FID method, with the use of a capillary column SPB-1701 30 m in length by means of a constant helium flow through the column in the amount of 1 cm^3^/min. The injector temperature was 250 °C and the detector’s was 280 °C. The temperature programme of the oven began at 40 °C and was kept for 2 min; the temperature then rose at a rate of 7 °C per minute, up to 100 °C. The final temperature of the programme (100 °C) was maintained for 1 min. The detection limit was 2.0–3.5 μg for a single compound. In order to make the calibration curve, the LGC Promochem formulas were used with an initial concentration of 200 μg/mL for each single compound. Recovery of a single compound varied from 89 to 92% (cumene) to 68–75% (benzene). The total sorption capacity was assessed by the weighing method (Wang et al. 2012). Two mathematical models were used to describe the kinetics—Lagergren’s and Ho-McKay’s (Dąbek 2015). Porosimetric investigations (sorption surface and pore volumes) were carried out with the sorption analyser ASAP 2420 M (Micromeritics). The statistical calculation (regression analysis) was conducted by means of the Statistica 12 package.

## Results and Discussion

The hydrocarbons studied are considered to be sparingly soluble in water and are usually viewed as a group of compounds which create a LNAPL (light non-aqueous phase liquid) layer after crossing the water environment. Nevertheless, the hydrocarbons’ properties allowed them to migrate into the aquatic phase (Table 1). The obtained concentrations of the compounds examined (except when using ethylene glycol) were lower than the maximal ones possible for water at 20 °C. When taking into account the solubility of the individual compounds, one can assume that they were dissolved in water and did not appear in the form of an emulsion. The concentrations obtained were very similar to the ones obtained in previous studies (Ciesielczuk et al. 2018 unpublished data). The examined sorbents—the broadleaf cattail seeds, and all of them reached from a homogenous material—appeared to be different from each other in terms of apparent density and maximal sorptivity. The biggest apparent density (Table 2) was noted for materials N and Na as submitted for the NaOH activity. The lowest one, on the other hand, was noted for H and the approximate G and S, which are characteristic for seeds adapted to wind propagation and similar in density to sisal and cotton fibres (Annunciado et al. 2005; Kim and Netravali 2010). The apparent density was rising along with the intensification of the mercerisation process in the range H < G < S < W < Na < N. A significant hydrophobicity of the analysed materials (without shaking, seeds are flowing on the water at least 7–10 days) makes the contact between the seed’s surface and an adsorbate more difficult. However, in the presence of a LNAPL layer, this phenomenon could be very useful. Maximal sorptivity (26.61 g/g) was noted for the G material which showed the lowest sorptive surface and a low mass of absorbed BTEX from a water solution. For the H and W seeds, despite applying mercerisation with hot water, only non-significantly better results for pour hydrocarbons were obtained. The BTEX sorption from a compound—especially for the W type—at 97.02 mg/g, which constituted 109.0% of the value, was reached for the S control which was definitely run much better. For the Na type, despite the similar BET surface, a three times lower total capacity and almost the lowest amount (78.98 mg/g) of the hydrocarbons absorbed from a solution were obtained. This might have been caused by a partial disintegration of wax fibres surfacing during the NaOH mercerisation which took 48 h and had high bulk density. In analogous studies carried out by other authors regarding the gas oil sorption of black poplar seeds, over 100 g/g of the total sorptive capacity was reached, pointing to the possibility of using them for a reduction in oil-related products’ bottling (Likon et al. 2013). The low density of the broadleaf cattail seeds examined here may lead to certain problems when using them for sorption purposes as their capability to be lifted up causes loss of the material when used under the influence of wind (Półka et al. 2015).

**Table 2:** Characteristic of sorbents used in experiments

Weed sample	C	H	W	N	Na	G
Bulk capacity (g/dm^3^)	1.435 (0.024)	1.417 (0.071)	1.798 (0.110)	2.352 (0.213)	2.301 (0.112)	1.422 (0.087)
Sorption capacity [g/g]	22.65	23.24	24.95	3.001	7.769	26.61
BET sorption surface [m^2^/g]	0.19	0.29	0.34	0.58	0.30	0.11
Langimur sorption surface [m^2^/g]	0.25	0.39	0.46	0.83	0.41	0.13
Total pore volume [mm^3^/g]	0.585	0.725	0.865	1.892	0.843	0.377
Mass of adsorbed hydrocarbons from solution *t* = 360 min. [mg/g]	88.96	89.54	97.02	76.49	78.98	82.20
Sorption change [%]	100.0	100.6	109.0	86.0	88.8	92.4

The fibrous materials of plant origin (corn and soya waste), after mechanical treatment and mercerisation with a hydroxide soda, constitute good sorbents. The sorptive capacity defined for oil-burning products is 450–500% m/m (Wong et al. 2016). In individual studies, broadleaf cattail seeds mercerised with NaOH (N, Na) have shown a sorptive capacity of 300–770%, which was the lowest value; the highest value was noted for the W and G seeds (2495–2661%). This allows it to be concluded that NaOH can be used for mercerisation of dense materials only (e.g. corn stems). What is more, applying this processing technique does not increase the capacity of natural fluffy materials, for example of broadleaf cattail seeds. Twice larger amounts (40–50 g/g) of the absorbed oil-related products were noted after the use of kapok fibres (*Ceiba pentandra L.)* (Wang et al. 2012). The ones obtained for the examined amounts of total sorptive capacity were bigger (except for the N type) than the ones noted for expanded perlite, cellulose fibres and sorbent from polypropylene which absorbed the biggest amount of the oil-related product, but which was still 8 times less than the noted capacity of poplar seeds (Likon et al. 2013). Much larger amounts (nearly 100 g/g, and 85 g/g after 24 h) were noted when silk fibres were applied as a sorbent; however, their application is dependent on the accessibility of waste from textiles (Annunciado et al. 2005).

In the course of studies focused on sorption and carried out by the static method (batch), it was shown that the amount of absorbed aromatic pollutions is dependent on the contact time of the polluted water and a sorbent as well as on the type of the mercerisation technique applied. In all cases, however, the process of mercerisation was flowing quite slowly, and no concentration balance was noted in any of the ones studied.

Sorption curves are better suited to a pseudo second-order equation with a correlation coefficient between 0.987–0.998 (in contrast to a pseudo first-order equation in which *R*^2^ is 0.912–0.994). Sorption speed (k1 and k2 factors) calculated with Lagergren’s and Ho-McKay’s models show that sorption speed depends on the seed’s surface modification and compound affinity (Tables 3 and 4).

**Table 3:** Pseudo first-order rate constant k1 for sorption of BTEX on analysed seeds

	C	H	W	N	Na	G
Benzene	0.0063	0.0049	0.0051	0.0047	0.0051	0.0047
Toluene	0.0043	0.0055	0.0048	0.0047	0.0047	0.0053
Ethylbenzene	0.0044	0.0039	0.0041	0.0043	0.0046	0.0061
p-Xylene	0.0037	0.0039	0.0046	0.0033	0.0058	0.0035
o-Xylene	0.0036	0.0048	0.0039	0.0041	0.0044	0.0050
Cumene	0.0032	0.0029	0.0037	0.0033	0.0034	0.0037

**Table 4:** Pseudo second-order rate constant k2 for sorption of BTEX on analysed seeds

	C	H	W	N	Na	G
Benzene	1.33E-05	5.00E-04	8.33E-06	1.56E-05	7.46E-06	9.96E-06
Toluene	2.13E-05	1.10E-03	1.41E-05	2.95E-05	2.00E-05	1.95E-05
Ethylbenzene	6.68E-05	3.20E-03	7.16E-05	5.46E-05	3.63E-05	3.19E-05
p-Xylene	6.17E-05	3.20E-03	5.26E-05	4.88E-05	9.17E-05	7.52E-05
o-Xylene	5.14E-05	3.40E-03	3.93E-05	1.34E-04	2.83E-05	2.47E-05
Cumene	2.45E-04	8.60E-03	2.40E-04	1.52E-04	7.46E-06	1.20E-04

**Fig. 1:**
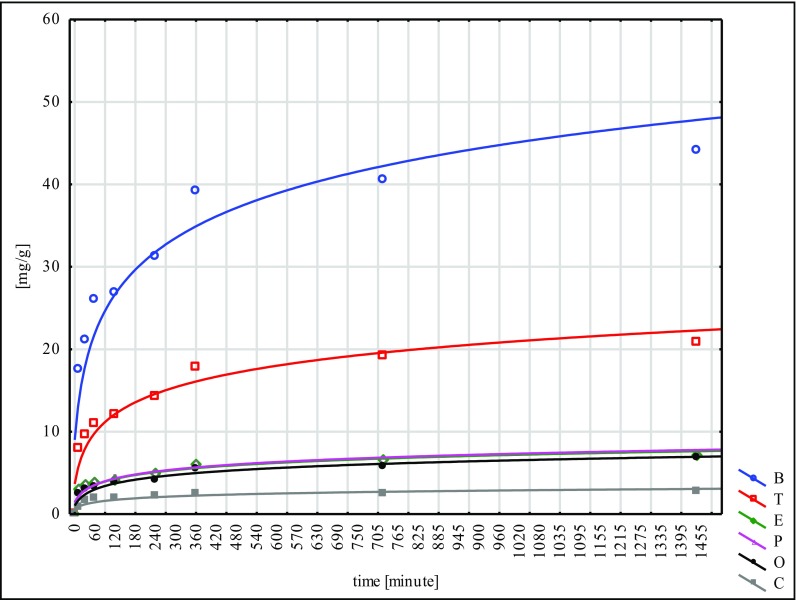
BTEX sorption on C weeds

**Fig. 2:**
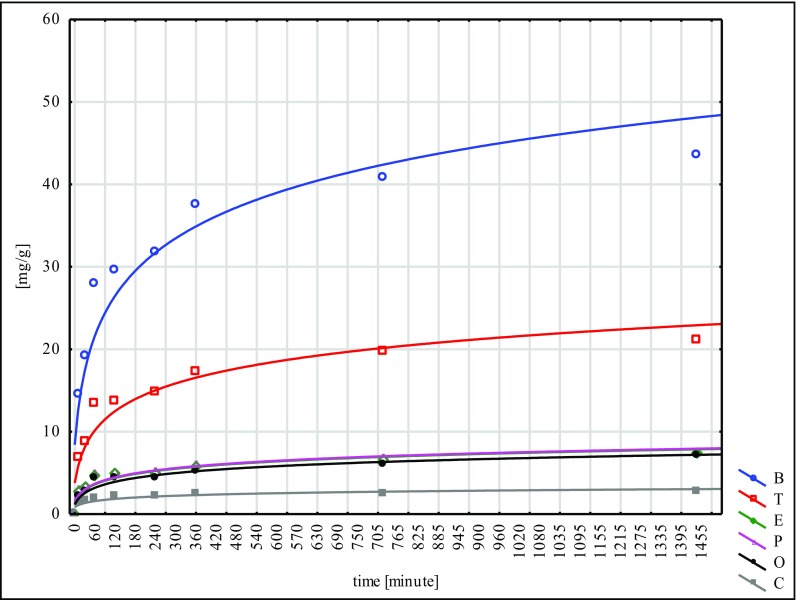
BTEX sorption on H weeds

**Fig. 3:**
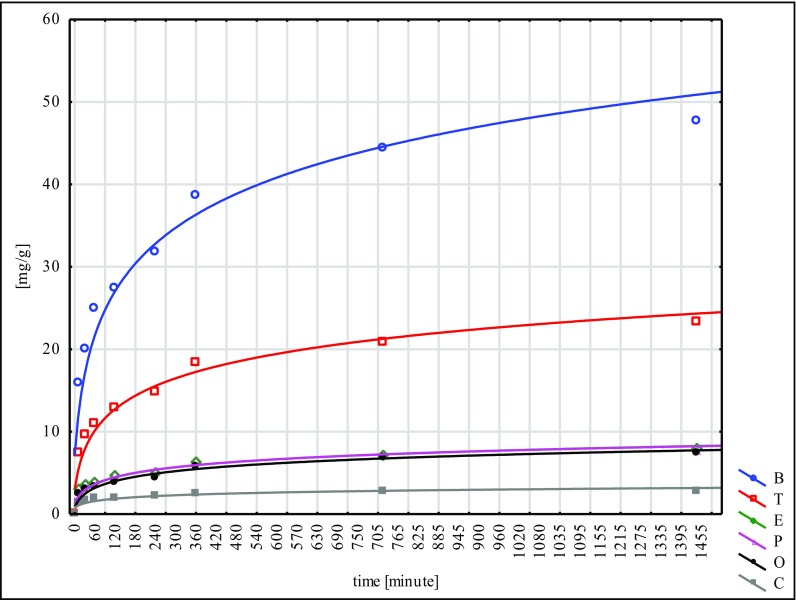
BTEX sorption on W weeds

**Fig. 4:**
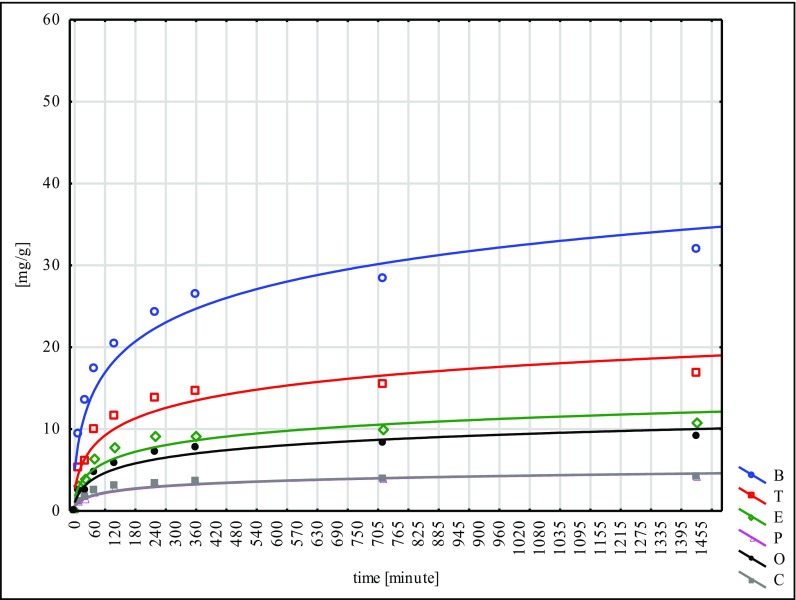
BTEX sorption on N weeds

**Fig. 5:**
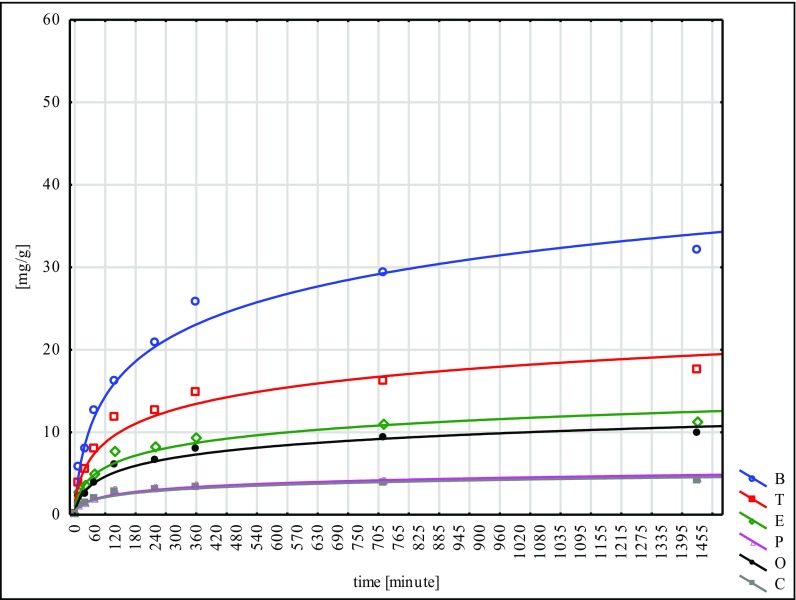
BTEX sorption on Na weeds

**Fig. 6:**
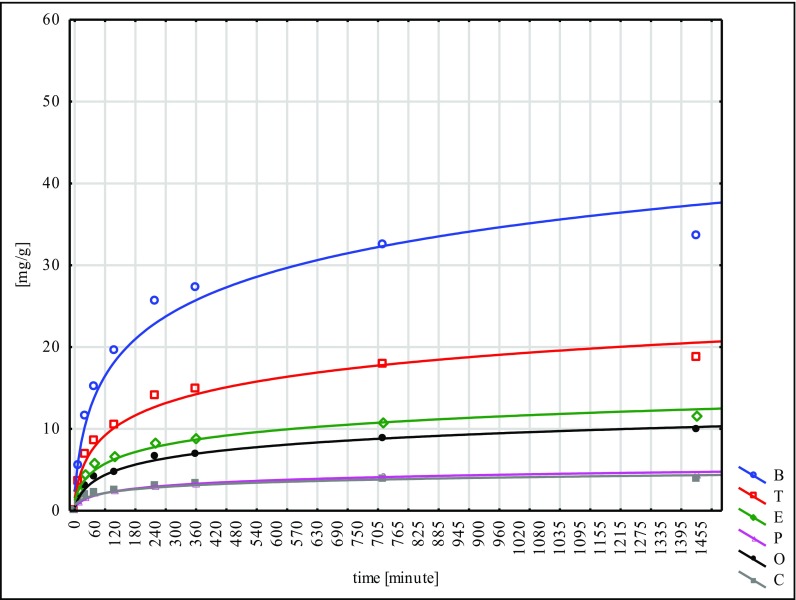
BTEX sorption on G weeds

The largest quantity of hydrocarbons was absorbed by the W material and the smallest by N. This fact points to the inconsiderable effectiveness of the NaOH mercerisation for 60 min. In the sorption tests of the poplar seeds conducted under similar conditions, the best results were reached for the G type, while the worst ones were for the H type. These facts lead to the conclusion that mercerisation with the use of NaOH does not bring good results for any of the seeds of the species tested (Ciesielczuk et al. 2018 unpublished data). Sorption on the modified material C was flowing in a similar way to the so-called modified ones, submitted to different types of mercerisation (Figs. 1, 2, 3, 4, 5 and 6). With respect to all the seeds examined, as far as the intensity of sorption is concerned, the one with benzene took the first place and with toluene, the second place. The sorption isotherms for ethylbenzene, o- and p-xylene were very similar for the C, W and H materials. Then, for the N, Na and G types, the sorption isotherms obtained for p-xylene and cumene were nearly identical, but for ethylbenzene, they were different. This means that they were flowing in a different way in contrast to the seeds that had not submitted to the C modification and mercerised with hot water (H, W). Cumene appeared to be the compound that was sorbed most slowly. Its sorption intensity was proportional to the compound concentrations included in an adsorbate. The correlation coefficient between the surface sorption BET and Langimur as well as the amount of the sorbed hydrocarbons was 0.703–0.709.

In the 1440th minute of the experiment, no desorption was noted for any of the cases analysed. When talking about the materials submitted to analysis, for C, H and W, benzene was sorbed in the weakest way, and for N, Na and G, o-xylene and ethylbenzene. The low amount of sorbed benzene (even below 70% with a contact time of 24 h) is a natural phenomenon; however, little sorption of o-xylene and ethylbenzene, especially the one noted for the N type, probably results from a low compound affinity to the mercerised surface of seeds (Moura et al. 2011). The number of compounds sorbed from a solution was increasing along with a decrease in their solubility in water (Table 5). Only o-xylene is an exception here, which was sorbed on the level of 50.6–78.8% despite its poor solubility.

**Table 5:** Capacity of BTEX hydrocarbons removed from solution on time *t* = 1440 [%]

	C	H	W	N	Na	G
Benzene	65.47	65.91	71.85	67.17	67.24	69.78
Toluene	71.26	72.36	79.86	67.86	69.86	73.87
Ethylbenzene	77.48	80.25	85.41	53.35	58.57	62.46
p-Xylene	78.61	79.84	82.24	81.05	84.19	85.72
o-Xylene	72.25	73.75	78.79	50.56	58.74	58.79
Cumene	83.95	85.04	87.6	70.67	74.32	69.77

This confirms the data obtained for the poplar seeds (Ciesielczuk et al. 2018). The broadleaf cattail seeds that were studied absorbed cumene in the strongest way (S, H, W) and p-xylene (N, Na, G); in the last phase of the experiment (*t* = 1440), they absorbed 87.6% of the cumene and 85.7% of the p-xylene.

The W material sorbed the analysed hydrocarbons a lot more effectively than seeds mercerised in a different way. In the end, this method appeared to be the most effective for a sorption capacity increase. This fact points to the dissimilar structure of the broadleaf cattail seeds’ fibres as they differ from the poplar seeds fibres for which drenching in cold water for a period of 15 days (G type) appeared to be the most effective technique of mercerisation (Ciesielczuk et al. 2018 unreleased data). A significant number of hydrocarbons absorbed from the water solution by means of the seeds tested can be explained by their huge pappus and their nanotube structure (Likon et al. 2013). In a similar experiment carried out with montmorillonite as a sorbent (Nourmoradi et al. 2012), a sorption of BTEX on the level of almost 19 mg/g was reached; this represents only 20% of the amounts obtained in the studies described above. The sorption of the broadleaf cattail seeds was effective; nevertheless, their considerable degree of hydrophobicity is a kind of an obstacle here as it inhibits the contact between a sorbent and an adsorbate. The examined hydrocarbons were sorbed in huge amounts, exceeding even the effects obtained by other authors when using ground peat, organo-silicate polymer and montmorillonite (Ciesielczuk 2017; Moura et al. 2011; Nourmoradi et al. 2012). The regression that was conducted showed a highly significant correlation (*p* < 0.01) between the sorption isotherms BTEX reached for differently mercerised broadleaf cattail seeds. It is confirmation of little change as far as the sorption of aromatic hydrocarbons dissolved in water with the usage of seeds submitted to mercerisation by the different methods concerned.

## Conclusions

As a result of the laboratory experiment conducted, the differential sorptive properties of mercerised broadleaf cattail seeds have been stated (*Typha latifolia L.*). The sorbents studied brought about a visible decrease of monoaromatic hydrocarbons concentrations in water; therefore, they might be used as means of limiting the effects of oil-related pollution penetration in water environments. Depending upon the mercerisation method applied, for the proportion of the seeds that were not submitted to mercerisation, a sorption rate of 86.0–109.0% was reached. The lowest sorptive properties were shown by seeds that were submitted to short and long-lasting NaOH mercerisation (N and Na). The best properties were seen in the W material, which points to the possibility of increasing the sorptive capacity of the broadleaf cattail seeds by drenching them in hot water for 4 h. Nonetheless, one should assess the costs of such a process, in view of the relatively good results obtained by applying broadleaf cattail seeds without mercerisation (C). An additional advantage supporting the idea of using the non-mercerised material (C) as a sorbent is the option of applying a cheap source of a fibrous organic matter that is accessible almost everywhere which has a moderate climate. After a contact time of 24 h with the W material, nearly 72% of easily soluble benzene and over 87% of cumene were removed. The amount of the sorbed substances can be ranged in the following way: cumene > ethylbenzene > p-xylene > toluene > o-xylene > benzene > all types of sorbents including non-mercerised seeds and controls. One should pay attention, however, to a different kind of process of sorption for the N, Na and G types. The significant hydrophobicity of the analysed materials makes the contact between the seed’s surface and an adsorbate more difficult, thus extending the time for the process. In the case of a hydrocarbon layer present on the water table, however, this could have a positive meaning.
